# The Juvenile-Hormone-Responsive Factor *AmKr-h1* Regulates Caste Differentiation in Honey Bees

**DOI:** 10.3390/biom13111657

**Published:** 2023-11-17

**Authors:** Zhi-Xian Gong, Fu-Ping Cheng, Jia-Ning Xu, Wei-Yu Yan, Zi-Long Wang

**Affiliations:** 1 Honeybee Research Institute, Jiangxi Agricultural University, Nanchang 330045, China; ling15350309823@stu.jxau.edu.cn (Z.-X.G.); cfp25@stu.jxau.edu.cn (F.-P.C.); ahonsz@stu.jxau.edu.cn (J.-N.X.); weiyuyan@jxau.edu.cn (W.-Y.Y.); 2 Jiangxi Province Key Laboratory of Honeybee Biology and Beekeeping, Nanchang 330045, China

**Keywords:** juvenile hormone, *AmKr-h1*, caste differentiation, RNAi, RNA-seq

## Abstract

Honey bees are typical model organisms for the study of caste differentiation, and the juvenile hormone (JH) is a crucial link in the regulatory network of caste differentiation in honey bees. To investigate the mechanism of JH-mediated caste differentiation, we analyzed the effect of the JH response gene *AmKr-h1* on this process. We observed that *AmKr-h1* expression levels were significantly higher in queen larvae than in worker larvae at the 48 h, 84 h, and 120 h larval stages, and were regulated by JH. Inhibiting *AmKr-h1* expression in honey bee larvae using RNAi could lead to the development of larvae toward workers. We also analyzed the transcriptome changes in honey bee larvae after *AmKr-h1* RNAi and identified 191 differentially expressed genes (DEGs) and 682 differentially expressed alternative splicing events (DEASEs); of these, many were related to honey bee caste differentiation. Our results indicate that *AmKr-h1* regulates caste differentiation in honey bees by acting as a JH-responsive gene.

## 1. Introduction

In honey bee colonies, the queen and worker are diploids, developing from fertilized eggs and, although they share the same genetic material, they differ significantly in morphological characteristics, behavior, and longevity [1]. Currently, nutrition [2], epigenetic modifications [3], and hormones [4] are considered to be important factors in regulating the differentiation of queen–worker castes. Of them, juvenile hormone (JH) is known to play a key role in this process [5].

JH is a pleiotropic hormone secreted by the insect corpora allata (CA) and released into the hemolymph [6]. It interacts antagonistically with 20-hydroxyecdysone (20E) to maintain larval traits in insects, where 20E induces molting and metamorphosis, and JH prevents the metamorphosis caused by 20E. During the last larva instar, the JH titer drops sharply, resulting in the pupation of completely metamorphosed insects and the emergence of incompletely metamorphosed insect adults [7,8,9,10]. In the honey bee, JH is a central regulator of the non-genetic diversity of social castes. Its physiological role in the honey bee is not only limited to maintaining the juvenile state but also has a significant ability to regulate growth and development, caste differentiation, and affects longevity [11,12]. Queen larvae have larger corpora allata and significantly higher JH titres in their hemolymph compared to worker larvae, especially at the critical point of caste differentiation, where the JH titre threshold determines the developmental trajectory of the larvae (development into a queen or worker). This suggests that caste differentiation is accompanied by strict regulation of JH synthesis and metabolism [5]. In addition, JH also regulates the synthesis of *vitellogenin* (*Vg*), which promotes ovarian development in honey bees, resulting in higher levels of Vg and more ovarian tubes in queens [13,14].

*Krüppel homolog 1* (*Kr-h1*), a C2H2 zinc finger (Znf) transcription factor, is an early responder gene for JH that can be activated by the JH receptors Met and Gce in *Drosophila* [9]. It plays a crucial role in the JH signaling pathway by regulating the expression of its downstream target genes. In many insects, the JH response elements (JHREs) in the *Kr-h1* promoter have been identified as a canonical E-box (CACGTG) or a C-box (CACGCG) [15,16,17], which are the typical binding elements for bHLH-PAS proteins and are also essential for JH-induced gene expression via Met [18,19]. The *Kr-h1* is highly expressed in the larval stage in insects and decreases in the pupal stage, which is consistent with changes in JH titres in haemolymph [20,21,22]. The main function of *Kr-h1* is to repress the metamorphosis of insects by transmitting JH signaling. It also regulates insects’ yolk production and egg maturation [23,24,25], but the regulatory effect of *Kr-h1* on yolk production varies across species. For example, down-regulation of *Kr-h1* in *Locusta migratoria* reduced the mRNA expression level of Vg by an average of 95%, which significantly inhibited oocyte maturation and ovarian development [26]. However, in *Tribolium castaneum* the low expression of *Kr-h1* could only down-regulate Vg by about 30% [27]. In addition, *Kr-h1* is also involved in the development of the nervous system of *Drosophila* larvae [28] and foraging behavior of honey bees [29].

In the JH signaling pathway, *Kr-h1* is downstream of the JH receptors Met and Gce and can be up-regulated by JH [30,31]. Due to the antagonistic effect of JH and 20E, *AmKr-h1* can inhibit 20E-induced metamorphosis by transducing JH signaling to suppress the expression of the major 20E response genes during the larval stage when JH titres are high [7,32]. At the terminal instar of insect larvae, the JH titre decreases dramatically and low-expressing *AmKr-h1* is unable to override high 20E signaling, thus, 20E-induced metamorphosis occurs [9].

Since *AmKr-h1* is regulated by JH, we speculate that *AmKr-h1* may be the key regulator in the queen–worker caste differentiation. Therefore, in the present study, we analyzed the function of *AmKr-h1* in the caste differentiation in honey bees using RNAi, and analyzed transcriptome changes in the bee larvae after *AmKr-h1* RNAi.

## 2. Materials and Methods

### 2.1. Insects

The honey bee colonies (*Apis mellifera ligustica*) used in this study were bred at the Honeybee Research Institute of Jiangxi Agricultural University (28.46 N, 115.49 E), Nanchang, Jiangxi, China.

### 2.2. Sequence Analysis of AmKr-h1 Gene

The exon–intron structure was analyzed by mapping the mRNA sequence (GenBank accession no.: NM_001242470.1, NM_001011566.1) of the *AmKr-h1* to the *A. mellifera* genome at the NCBI website from the *A. mellifera AmKr-h1* gene deposited in the GenBank database (https://www.ncbi.nlm.nih.gov/genbank/, accessed on 20 March 2022). The structural domains of the *AmKr-h1* protein were analyzed at the SMART online website (http://smart.embl-heidelberg.de/, accessed on 20 March 2022). Multiple sequence alignments of amino acid sequences downloaded from NCBI were performed using the CLUSTALX v1.81 software [33].

### 2.3. Expression Analysis Using qRT-PCR

A queen was restricted on an empty comb to lay eggs for six hours. After the eggs hatched into larvae, some of the larvae were transferred to queen cells, while the remaining larvae continued to be bred in worker cells. Six biological replicates of both queen larvae and worker larvae were sampled at 48 h, 84 h, and 120 h after hatching of the eggs for qRT-PCR analysis. During sampling, the larvae were washed three times with double-distilled water to remove any royal jelly, and then were stored in liquid nitrogen for further use.

Total RNA was extracted from the collected samples using the TrizolUp Kit (Transgen, ER501-01, Beijing, China), and the RNA concentration of each sample was measured using a spectrophotometer. A total of 1 µg of RNA was taken from each sample and reverse transcribed into cDNA using M-MLV reverse transcriptase (TakaRa, RR047A, Dalian, China). The *Gapdh* (GenBank accession no.: NM_001014994.1) gene was used as the internal reference. 

The qRT-PCR primer sequences of each gene were designed using the Primer Premier 5.0 software based on their mRNA sequences (Table S1). The qRT-PCR reaction system was as follows: 5.0 µL of SYBR GREEN, 0.2 µL of ROX correction fluid, 0.4 µL of forward primer (0.0125 nmol/μL), 0.4 µL of reverse primer (0.0125 nmol/μL), 1.0 µL of cDNA (150 μg/μL), and 3.0 µL of ddH2O. The qRT-PCR amplification conditions were as follows: 95 °C for 10 min; followed by 40 cycles of 95 °C for 15 s, Tm for 1 min. Four technical replicates were set up for each biological replicate. The data were analyzed using the 2^−ΔΔCT^ method [34] and *t*-tests were performed using the SPSS 26.0 software to analyze the differences in gene expression.

### 2.4. JH Treatment

The queen of an *A. mellifera* colony was controlled on an empty comb to lay eggs for 6 h. When the eggs hatched, the larvae were transferred to a 24-well culture plate containing 300 µL of artificially prepared food (6% fructose, 6% glucose, 1% yeast, 37% distilled water, and 50% royal jelly) per well. The larvae were randomly divided into three groups: to the first group (JH), 1 µL of juvenile hormone III (JH III) (APExBIO, purity ≥ 65.00%) solution diluted with ethanol (10 µg/µL) was dripped on the back; the second group (ET) received 1 µL of ethanol on the back as a control; the third group (NO) was normal larvae that received no treatment. Then, the larvae were moved to an incubator at 34 °C and 85% humidity. For the next 2 days, the JH group and ethanol group continued to be treated with JH III or ethanol three times per day. Meanwhile, each group received 150 µL of artificial foods twice daily. 

Twenty-four hours after treatment completion, the larvae in each group were collected for mRNA expression analysis using qRT-PCR. For each treatment group, seven biological replicates with two larvae per replicate were sampled. The samples were washed three times with double-distilled water and immediately snap-frozen in liquid nitrogen, and then stored in liquid nitrogen for RNA extraction. The qRT-PCR protocol detailed in Section 2.3 was followed. The differences in the expression of *AmKr-h1* between the JH and two control groups were analyzed by ANOVA using SPSS 26.0.

### 2.5. RNAi Treatment

The siRNA sequences of *AmKr-h1* and the control (Con) were as follows: *AmKr-h1*, sense: GGUACAUACGCGUACGCAUTT, antisense: AUGCGUACGCGUAUGUACCTT; Con, sense: UUCUCCGAACGUGUCACGUTT, antisense: ACGUGACACGUUCGGAGAATT. The siRNA was synthesized by Genepharma (Shanghai, China).

Once again, a queen of an *A. mellifera* colony was limited to an empty comb to lay eggs for 6 h. After hatching of the eggs, the one-day-old larvae were transferred to a 24-well culture plate containing 300 µL of artificially prepared food in each well. The plates were kept in an incubator at 34 °C and 85% humidity. Then, the larvae were fed twice per day with 150 μL of artificial food. When they reached 60 h, the larvae were randomly divided into two groups and injected with the *AmKr-h1* siRNA solution (solubilizing siRNA with RNase-free water; the concentration was 2 μg/μL) and the NT siRNA solution (solubilizing Con with RNase-free water; the concentration was 2 μg/μL), respectively. The NT group was set as the control group. Each larva was injected with 400 ng of siRNA and then provided with 300 µL of artificial food and kept in an incubator (34 °C, 85% humidity). When the larvae were 3 days old, they were transferred to a new plate and fed twice per day with 200 µL of artificial food until pupation.

At 48 h after manual injection of siRNA, ten surviving larvae were selected and set as five biological replicates (each containing two larvae) for detecting the efficiency of RNAi by qRT-PCR. Total RNA extraction, cDNA synthesis, and qRT-PCR were performed as described above. The remaining larvae were kept in the incubator until emergence. The morphological traits of 35 newly emerged bees from the Con group and 35 bees from the *AmKr-h1* RNAi group were measured as follows. Firstly, bees were frozen to death on ice. Then, the body weight of each bee in each group was weighed with an electronic balance, and the length of the body, proboscis, and forewing of each bee was measured by a stereomicroscope (Guiguang, GL-99TI, Guilin, China) with the tiny creature morphological measurement and data analysis system of the Optec OPTPro software (cnoptec, version: x86, Chongqing, China). All data were analyzed by *t*-test in SPSS 26.0.

### 2.6. cDNA Library Construction and Sequencing

Total RNA was extracted from both the RNAi group and Con group samples (each sample containing two larvae). After measuring the quality and concentration of the RNA samples, the mRNA molecules were enriched using Oligo (dT) magnetic beads, and then fragmentation buffer was added to break them randomly into small fragments. Using these small fragments as templates, double-stranded cDNA was synthesized by reverse transcriptase. Then, cDNA fragments were purified, end-repaired, and poly(A) tails and Illumina sequencing adaptors were added. The cDNA fragments were isolated using AMPure XPbeads and were PCR-amplified, the PCR products were purified again to build a cDNA sequencing library. Finally, the cDNA libraries were sequenced using the Illumina high-throughput sequencing platform.

The raw sequences (raw reads) obtained from sequencing were filtered with the fastp software by removing adaptors, N (no base information could be determined) ratio > 10%, low-quality (the number of bases with quality value Q ≤ 10 accounted for more than 50% of the whole read), and trimmed sequences < 500 bp in length, to obtain clean sequences (clean reads). Clean reads were compared with the *A. mellifera* reference genome sequences (https://ftp.ncbi.nlm.nih.gov/genomes/all/GCF/003/254/395/GCF_003254395.2_Amel_HAv3.1/, accessed on 19 January 2022).

### 2.7. Screening of DEGs and DEASEs

The expression level of each gene was calculated by counting the number of reads matched to each gene with the Cufflinks v2.2.1 software. Differentially expressed genes were screened by DESeq using ∣log2(fold change)∣ > 2 and *p*-value < 0.05 as the criteria. Then, a GO and KEGG enrichment analysis was performed with the DEGs. Differentially expressed alternative splicing events were analyzed using the rMATS v4.1.2 software with FDR < 0.05 as a screening criterion. The reliability of the RNA-seq results of nine DEGs was verified using qRT-PCR, as mentioned above.

## 3. Results

### 3.1. AmKr-h1 Encodes a Zinc Finger Protein

**Figure 1:**
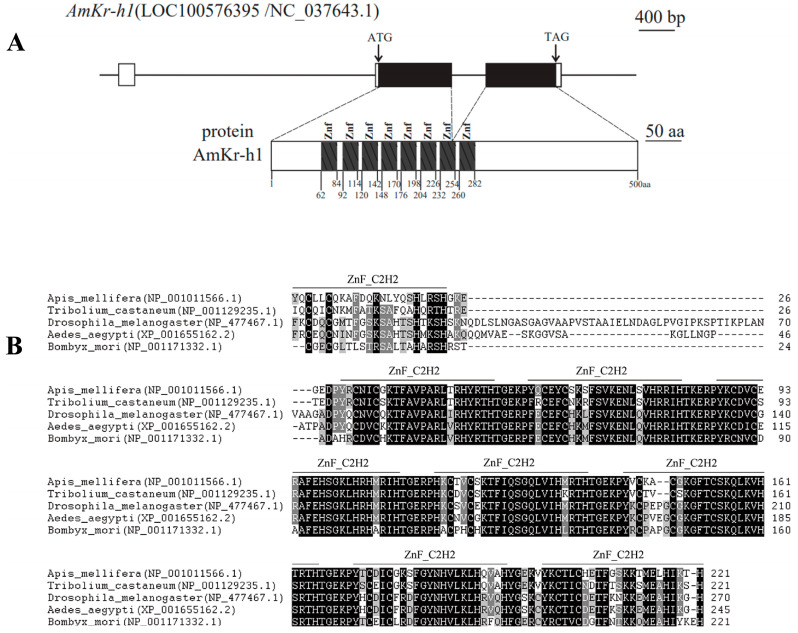
The intron/exon structure and amino acid sequence comparison of the *AmKr-h1* gene of insects. (**A**) The intron/exon structure and predicted protein of *AmKr-h1* gene. In the upper part, boxes, lines, and black boxes represent exons, introns, and coding regions, respectively; (**B**) Amino acid sequence comparison of the C_2_H_2_ zinc finger domains between the *Kr-h1* protein of *A. mellifera* and other species.

The mRNA sequence of *A. mellifera AmKr-h1* is 1756 bp long, containing three exons and encoding a protein of 500 aa, containing eight C2H2 zinc finger (ZnF_C2H2) domains (Figure 1A). The *AmKr-h1* protein is highly conserved in the ZnF_C2H2 domain region compared with those of *Aedes aegypti*, *T. castaneum*, *Drosophila melanogaster*, and *Bombyx mori* (Figure 1B).

### 3.2. AmKr-h1 Has Higher Expression in Queen Larvae and Is Regulated by JH

**Figure 2:**
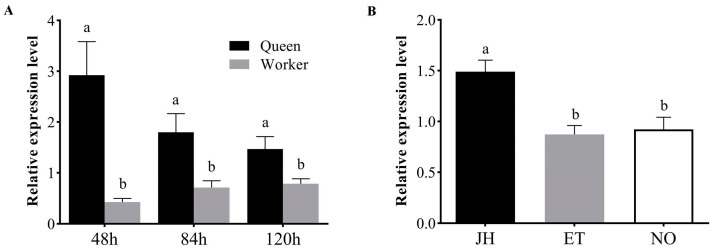
Differences in expression of *AmKr-h1* between *A. mellifera* queen larvae and worker larvae as well as expression changes after JH treatment. (**A**) Relative mRNA expression levels of *AmKr-h1* in queen larvae and worker larvae at 48 h, 84 h, and 120 h of the larval stage. Different lowercase letters represent significant differences at *p* < 0.05; the same in (**B**). (**B**) Relative mRNA expression levels of *AmKr-h1* in larvae of *Apis mellifera* after JH treatment. JH: the group treated with juvenile hormone III; ET: the group treated with ethanol; NO: the group without treatment.

The relative mRNA expression levels of *AmKr-h1* between queen larvae and worker larvae at 48 h, 84 h, and 120 h after the hatching of eggs were measured using quantitative real-time polymerase chain reaction (qRT-PCR). The results showed that the expression levels of the *AmKr-h1* gene in the queen larvae were significantly higher than in the worker bee larvae at all the three time points (*t*-test, *p* < 0.05) (Figure 2A).

We investigated the effect of JH on the expression of *AmKr-h1*. After treatment of the larvae with JH, the expression level of *AmKr-h1* in the JH group was significantly higher than that in the ethanol group and the untreated group (ANOVA, *p* < 0.05), and there was no significant difference between the ethanol group and the no treatment group (ANOVA, *p* > 0.05) (Figure 2B).

### 3.3. AmKr-h1 Affects Castes Differentiation of Queen–Worker

**Figure 3:**
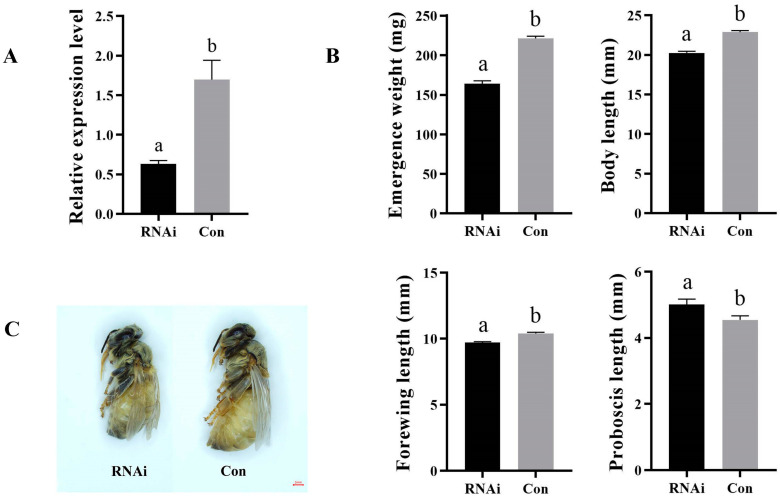
Effect of *AmKr-h1* on honey bee caste differentiation. (**A**) Expression change of *AmKr-h1* in larvae of *A. mellifera* after RNAi. Different lowercase letters represent a significant difference at *p* < 0.05; the same in (**B**). (**B**) Morphological changes in newly emerged bees after *AmKr-h1* RNAi. (**C**) An example of morphological change in newly emerged bees after *AmKr-h1* RNAi. “Con” represents control group.

The expression changes in *AmKr-h1* after RNAi were analyzed using qRT-PCR, and the results showed that the expression levels of *AmKr-h1* in the RNAi group were significantly lower than in the Con group (*t*-test, *p* < 0.05) (Figure 3A). This indicated that our RNAi experiment was effective.

The morphological indexes of the newly emerged adult bees in the RNAi and the Con groups were measured. After knocking down the expression of *AmKr-h1*, the emergence weight, body length, and forewing length of the newly emerged bees in the RNAi group decreased significantly compared with the Con group (*t*-test, *p* < 0.05), while the proboscis length was significantly longer than in the Con group (*t*-test, *p* < 0.05) (Figure 3B). The bees in the RNAi group were significantly smaller than those in the Con group (Figure 3C). This indicates that reduced *AmKr-h1* expression can lead to the differentiation of larvae into workers.

### 3.4. Summary of Transcriptome Sequencing Data

The number of raw reads obtained after sequencing for each biological replicate in the RNAi and Con groups ranged from 45,302,028 to 62,997,770, and the number of clean reads remaining after filtering low-quality reads and ambiguous reads ranged from 43,925,842 to 60,493,146. The percentages of Q20 and Q30 bases in each sample were higher than 97.16% and 92.36%, respectively. The GC content of clean reads in each sample was between 40.66 and 41.41%. The number of reads matched to the honey bee reference genome ranged from 41,673,370 to 57,479,542, with the proportion ranging from 89.45% to 95.28%, and the uniquely matched reads ranged from 40,781,360 to 56,370,089, with the proportion ranging from 87.76% to 93.83% (Table S2).

The correlation coefficients between biological replicates in the RNAi and Con groups were from 0.9388 to 0.9972, indicating high reliability between biological replicates (Table S3).

### 3.5. DEGs between the RNAi and Con Groups

**Figure 4:**
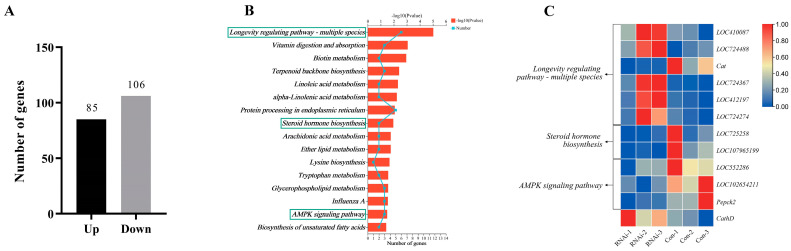
DEGs between the RNAi and Con groups. (**A**) Up- and down-regulated DEGs in the RNAi group compared to the control group in the larval stage of *A. mellifera*. Both the RNAi group and control group contain three biological replicates. (**B**) The significantly enriched KEGG pathways of the DEGs identified between the RNAi group and the control group. Pathways marked with a box are those associated with caste differentiation. (**C**) The DEGs involved in honey bee caste differentiation. “Con” represents control group.

A total of 191 DEGs were identified between the RNAi and Con groups. Among them, 85 genes were up-regulated and 106 genes were down-regulated in the RNAi group compared with the Con group (Figure 4A, Table S4).

The KEGG pathway enrichment analysis of the DEGs showed that 16 KEGG pathways were significantly enriched (*p*-value < 0.05), including “longevity-regulating pathway—multiple species”, “biotin metabolism”, “vitamin digestion and absorption”, “linoleic acid metabolism”, “terpenoid backbone biosynthesis”, “alpha-linolenic acid metabolism”, “protein processing in endoplasmic reticulum”, “steroid hormone biosynthesis”, “arachidonic acid metabolism”, “ether lipid metabolism”, “lysine biosynthesis”, “influenza A”, “glycerophospholipid metabolism”, “AMPK signaling pathway”, “tryptophan metabolism”, and “biosynthesis of unsaturated fatty acids”. Among these, the “longevity-regulating pathway—multiple species”, “steroid hormone biosynthesis”, and “AMPK signaling pathway” were associated with honey bee caste differentiation (Figure 4B, Table S5), including a total of eleven DEGs (Figure 4C), and the other pathways were mainly related to metabolism.

**Figure 5:**
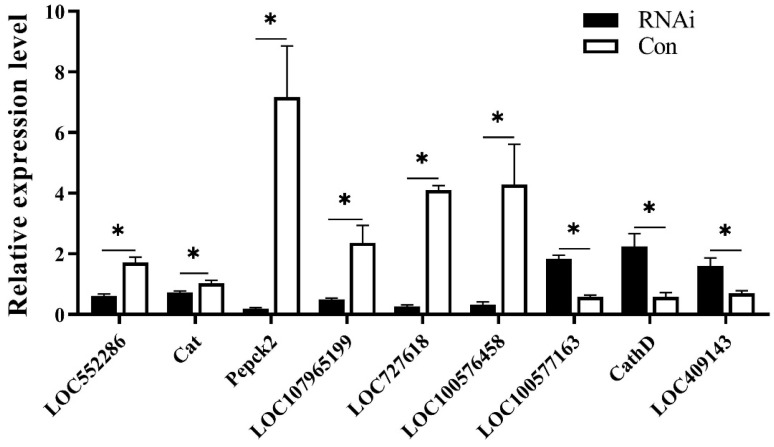
Verification of DEGs using qRT-PCR. Quantitative RT-PCR verification of nine DEGs between the RNAi and control (Con) groups. Asterisk (*) represents significant difference at *p* < 0.05.

Nine DEGs were selected for verifying the reliability of the RNA-seq results using qRT-PCR, including *LOC552286*, *Catalase* (*Cat*, *LOC443552*), *Phosphoenolpyruvate carboxykinase 2* (*Pepck 2*, *LOC412843*), *LOC107965199*, *LOC727618*, *LOC100576458*, *LOC100577163*, *Cathepsin D* (*CathD*, *LOC409341*), and *LOC409143.* The results indicated that all of them were significantly differentially expressed between the RNAi group and the control group, while six of these genes were down-regulated and three genes up-regulated in the RNAi group. These results are consistent with the RNA-seq results, indicating that the transcriptome sequencing results are reliable (Figure 5).

### 3.6. DEASEs between the RNAi and Control Groups

**Figure 6:**
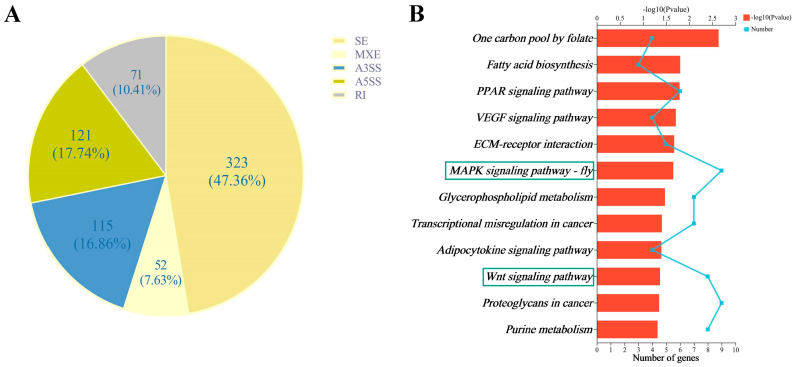
DEASEs between the RNAi and Con groups of honey bee. (**A**) Differential alternative splicing events between the RNAi group and control (Con) group. (**B**) The significant enriched KEGG pathways between the RNAi group and control (Con) group. The pathways marked with a box are associated with honey bee caste differentiation.

In this study, five alternative splicing forms between the RNAi and Con groups were analyzed, including skipped exon (SE), alternative 5′ splice site (A5SS), alternative 3′ splice site (A3SS), mutually exclusive exons (MXE), and retained intron (RI), and a total of 42,174 and 44,051 splicing forms were detected in the three biological replicates from the RNAi and Con groups, respectively, of which skipped exon (SE) was the most splicing type. In addition, there were 682 DEASEs related to 495 genes between the RNAi and Con groups, including 323 SE, 121 A5SS, 115 A3SS, 52 MXE, and 71 RI (Figure 6A), related to 262, 113, 101, 41, and 49 genes, respectively (Figure 6A, Table S6). Of the genes related to these DEASEs, *HR3* (*LOC408586*), *Hex110* (*LOC551648*), and *Br-C* (*LOC552255*) were involved in honey bee caste differentiation.

The KEGG pathway analysis of the DEASE-related genes revealed that a total of 12 KEGG pathways were significantly enriched (*p*-value < 0.05), including “one carbon pool by folate”, “fatty acid biosynthesis”, “PPAR signaling pathway”, “VEGF signaling pathway”, “ECM-receptor interaction”, “MAPK signaling pathway—fly”, “glycerophospholipid metabolism”, “transcriptional misregulation in cancer”, “adipocytokine signaling pathway”, “Wnt signaling pathway”, “proteoglycans in cancer”, and “purine metabolism” (Figure 6B, Table S7). Among these, the “MAPK signaling pathway—fly” and the “Wnt signaling pathway” were associated with honey bee caste differentiation.

## 4. Discussion

In the present study, we found that RNAi of the *AmKr-h1* gene resulted in the development of honey bee larvae into workers. In honey bees, JH is the principal factor influencing caste differentiation. *AmKr-h1* is the response gene downstream of the JH receptors Met and Gce, which are early response genes to JH [30,31]. Data from our results suggest that JH regulates the caste differentiation in honey bees by acting on the *AmKr-h1* gene.

We analyzed gene expression changes in honey bee larvae after *AmKr-h1* gene knockdown and identified many differentially expressed genes associated with honey bee caste differentiation, including *Cat* (*LOC443552*), *CathD* (*LOC409341*), and *Pepck2* (*LOC412843*).

The *Cat* gene encodes a catalase important to the cellular antioxidant system. It can guide the conversion of oxygen radicals in cells into hydrogen peroxide, with lower oxidative activity, and then be broken down, thus reducing the degree of cellular oxidation [35]. Studies have shown that *Cat* is significantly expressed at a higher level in queen larvae than in worker larvae, suggesting that the queen is more efficient in removing reactive oxygen species (ROS) and has a stronger antioxidant capacity than workers [36]. In our results, the expression of the *Cat* gene was down-regulated after *AmKr-h1* RNAi, suggesting that *AmKr-h1* may influence the antioxidant capacity of honey bees by regulating the *Cat* gene.

The *CathD* gene encodes a lysosomal aspartate protease. In insects, lysosome-mediated organelle degradation is particularly important in liposomal remodeling during the vitellogenesis cycle of female larvae [37,38]. In the fat bodies of insects with periodic egg maturation, lysosomes play a key role in terminating vitellogenesis by selectively degrading the secretory machinery involved in the mass production of yolk protein precursors [39]. During the termination of vitellogenesis in *A. aegypti*, the number of lysosomes in the fat body increases significantly [40]. In the present study, the expression of the *CathD* gene was up-regulated after RNAi of the *AmKr-h1* gene, indicating that *AmKr-h1* can suppress the expression of *CathD*, which leads to a decrease in aspartate protease activity and, thus, promotes honey bee ovarian development.

The *Pepck2* encodes a phosphoenolpyruvate carboxykinase (PEPCK) that is closely associated with pyruvate homeostasis [41]. In *Helicoverpa armigera*, high levels of pyruvate promote growth and development, and low levels of pyruvate lead to reduced metabolic activity [42]. In the present study, the expression of *Pepck2* was significantly down-regulated after RNAi of the *AmKr-h1* gene, indicating that the *AmKr-h1* gene may affect the growth and development of honey bees by regulating *Pepck2*.

Alternative splicing has a significant effect on caste differentiation [43]. Among the genes associated with DEASEs, we found three genes associated with honey bee caste differentiation: *HR3*, *Hex110*, and *Br-C*. The *HR3* gene encodes for a 20E-induced transcription factor that acts as a developmental switch in 20E-regulated insect development and metamorphosis [44]. In *A. aegypti*, inhibition of *HR3* gene expression by RNAi resulted in diminutive ovaries and a significantly reduced number of eggs [45]. Hexamerins are mainly involved in the dynamic changes in amino acid storage and utilization that occur during insect development and they can also function as JH-binding proteins [46,47,48]. In *Reticulitermes flavipes*, inhibition of hexamerins’ expression promotes the differentiation of larvae into workers [49]. The *Hex110* gene is highly expressed in the ovaries of the queens and is associated with the reproductive function in honey bees [50]. *Br-C*, a 20E response gene, is essential for maintaining the ecdysteroid titer [51]. During larval–pupal metamorphosis, Ecdysone signaling induces programmed cell death by inducing *Br-C* gene expression, eliminating larval tissues, and promoting adult tissue formation [52]. RNAi of the *Br-C* gene in *Drosophila* larvae at 120 h leads to a delay in pupal development [53]. In the present study, the expression levels of the *HR3* and *Hex110* isoforms were down-regulated and the *Br-C* isoform was up-regulated after inhibiting *AmKr-h1* expression, indicating that *AmKr-h1* may influence the caste differentiation in honey bees by positively regulating the expression levels of the *HR3* and *Hex110* isoforms while negatively regulating the *Br-C* isoform.

Through a KEGG pathway enrichment analysis of DEGs and DEASE-related genes, we found that several important pathways related to caste differentiation in honey bees were significantly enriched, such as the longevity-regulating pathway—multiple species, AMPK signaling pathway, MAPK signaling pathway—fly, and Wnt signaling pathway. This suggests that the JH signaling pathway has a cross-talk with the Wnt signaling pathway. It suggests that *AmKr-h1*, upon receiving juvenile hormone signals, may induce differentiation of queen and worker bees by acting on these critical pathways, but further experimental verification is needed.

## 5. Conclusions

In summary, our results indicate that JH regulates caste differentiation in honey bees by acting on *AmKr-h1*. Moreover, we identified a large number of differentially expressed genes after *AmKr-h1* knockdown, and future studies should focus on elucidating the specific pathways downstream of *AmKr-h1* that affect phenotypic differentiation between honey bee castes.

## Data Availability

The raw data of the RNA-seq are available from the NCBI SRA database, under accession number PRJNA996350.

## Supplementary Materials

The following supporting information can be downloaded at: https://www.mdpi.com/article/10.3390/biom13111657/s1, Table S1: Specific primers used for quantitative real-time PCR; Table S2: Summary of the transcriptome sequencing data; Table S3: The correlation coefficients between biological replicates; Table S4: DEGs between the RNAi and Con groups; Table S5: The significantly enriched KEGG pathways of the DEGs; Table S6: DEASEs between the RNAi and Con groups; Table S7: The significantly enriched KEGG pathways of the DEASESs.

## References

[1] Barchuk A.R., Cristino A.S., Kucharski R., Costa L.F., Simões Z.L., Maleszka R. (2007). Molecular determinants of caste differentiation in the highly eusocial honeybee *Apis mellifera*. BMC Dev. Biol..

[2] Kamakura M. (2011). Royalactin induces queen differentiation in honeybees. Nature.

[3] Kucharski R., Maleszka J., Foret S., Maleszka R. (2008). Nutritional Control of Reproductive Status in Honeybees via DNA Methylation. Science.

[4] Shuel R.W., Dixon S.E. (1960). The early establishment of dimorphism in the female honeybee, *Apis mellifera* L.. Insect. Soc..

[5] Hartfelder K., Engels W., Pedersen R.A., Schatten G.P. (1998). 2 Social Insect Polymorphism: Hormonal Regulation of Plasticity in Development and Reproduction in the Honeybee. Current Topics in Developmental Biology.

[6] Kotaki T., Shinada T., Kaihara K., Ohfune Y., Numata H. (2009). Structure Determination of a New Juvenile Hormone from a Heteropteran Insect. Org. Lett..

[7] Belles X., Santos C.G. (2014). The MEKRE93 (Methoprene tolerant-Krüppel homolog 1-E93) pathway in the regulation of insect metamorphosis, and the homology of the pupal stage. Insect Biochem. Mol. Biol..

[8] Jindra M., Bellés X., Shinoda T. (2015). Molecular basis of juvenile hormone signaling. Curr. Opin. Insect Sci..

[9] Li K., Jia Q.Q., Li S. (2018). Juvenile hormone signaling—A mini review. Insect Sci..

[10] Liu S., Li K., Gao Y., Liu X., Chen W., Ge W., Feng Q., Palli S.R., Li S. (2018). Antagonistic actions of juvenile hormone and 20-hydroxyecdysone within the ring gland determine developmental transitions in *Drosophila*. Proc. Natl. Acad. Sci. USA.

[11] Jindra M., Palli S.R., Riddiford L.M. (2013). The Juvenile Hormone Signaling Pathway in Insect Development. Annu. Rev. Entomol..

[12] Roy S., Saha T.T., Zou Z., Raikhel A.S. (2018). Regulatory Pathways Controlling Female Insect Reproduction. Annu. Rev. Entomol..

[13] Wirtz P., Beetsma J. (1972). Induction of caste differentiation in the honeybee (*Apis mellifera*) by juvenile hormone. Entomol. Exp. Appl..

[14] Chen S., Lin C., Lu K. (2012). cDNA isolation, expression, and hormonal regulation of yolk protein genes in the oriental fruit fly, *Bactrocera dorsalis* (Hendel) (Diptera: Tephritidae). J. Insect Physiol..

[15] Kayukawa T., Tateishi K., Shinoda T. (2013). Establishment of a versatile cell line for juvenile hormone signaling analysis in *Tribolium castaneum*. Sci. Rep..

[16] He Q., Wen D., Jia Q., Cui C., Wang J., Palli S.R., Li S. (2014). Heat Shock Protein 83 (Hsp83) Facilitates Methoprene-tolerant (Met) Nuclear Import to Modulate Juvenile Hormone Signaling. J. Biol. Chem..

[17] Chen X., Hu Y., Zheng H., Cao L., Niu D., Yu D., Sun Y., Hu S., Hu F. (2012). Transcriptome comparison between honey bee queen- and worker-destined larvae. Insect Biochem. Mol. Biol..

[18] Li M., Liu P., Wiley J.D., Ojani R., Bevan D.R., Li J., Zhu J. (2014). A steroid receptor coactivator acts as the DNA-binding partner of the methoprene-tolerant protein in regulating juvenile hormone response genes. Mol. Cell. Endocrinol..

[19] Kewley R.J., Whitelaw M.L., Chapman-Smith A. (2004). The mammalian basic helix–loop–helix/PAS family of transcriptional regulators. Int. J. Biochem. Cell Biol..

[20] Martín D., Chafino S., Franch-Marro X. (2021). How stage identity is established in insects: The role of the Metamorphic Gene Network. Curr. Opin. Insect Sci..

[21] Truman J.W. (2019). The Evolution of Insect Metamorphosis. Curr. Biol..

[22] Jindra M. (2019). Where did the pupa come from? The timing of juvenile hormone signalling supports homology between stages of hemimetabolous and holometabolous insects. Philos. Trans. R. Soc. B-Biol. Sci..

[23] Pecasse F., Beck Y., Ruiz C., Richards G. (2000). Krüppel-homolog, a Stage-Specific Modulator of the Prepupal Ecdysone Response, Is Essential for *Drosophila* Metamorphosis. Dev. Biol..

[24] Minakuchi C., Namiki T., Shinoda T. (2009). Krüppel homolog 1, an early juvenile hormone-response gene downstream of Methoprene-tolerant, mediates its anti-metamorphic action in the red flour beetle *Tribolium castaneum*. Dev. Biol..

[25] Lozano J., Belles X. (2011). Conserved repressive function of Krüppel homolog 1 on insect metamorphosis in hemimetabolous and holometabolous species. Sci. Rep..

[26] Song J., Wu Z., Wang Z., Deng S., Zhou S. (2014). Krüppel-homolog 1 mediates juvenile hormone action to promote vitellogenesis and oocyte maturation in the migratory locust. Insect Biochem. Mol. Biol..

[27] Parthasarathy R., Sun Z., Bai H., Palli S.R. (2010). Juvenile hormone regulation of vitellogenin synthesis in the red flour beetle, *Tribolium castaneum*. Insect Biochem. Mol. Biol..

[28] Fichelson P., Brigui A., Pichaud F. (2012). Orthodenticle and Kruppel homolog 1 regulate *Drosophila* photoreceptor maturation. Proc. Natl. Acad. Sci. USA.

[29] Fussnecker B., Grozinger C. (2008). Dissecting the role of Kr-h1 brain gene expression in foraging behavior in honey bees (*Apis mellifera*). Insect Mol. Biol..

[30] Abdou M., Peng C., Huang J., Zyaan O., Wang S., Li S., Wang J. (2011). Wnt Signaling Cross-Talks with JH Signaling by Suppressing Met and gce Expression. PLoS ONE.

[31] Bernardo T.J., Dubrovsky E.B. (2012). The *Drosophila* Juvenile Hormone Receptor Candidates Methoprene-tolerant (MET) and Germ Cell-expressed (GCE) Utilize a Conserved LIXXL Motif to Bind the FTZ-F1 Nuclear Receptor. J. Biol. Chem..

[32] Dubrovsky E. (2005). Hormonal cross talk in insect development. Trends Endocrinol. Metab..

[33] Thompson J.D., Gibson T.J., Higgins D.G. (2003). Multiple sequence alignment using ClustalW and ClustalX. Curr. Protoc. Bioinform..

[34] Schmittgen T.D., Livak K.J. (2008). Analyzing real-time PCR data by the comparative CT method. Nat. Protoc..

[35] Lei X.G., Zhu J., Cheng W., Bao Y., Ho Y., Reddi A.R., Holmgren A., Arnér E.S.J. (2016). Paradoxical Roles of Antioxidant Enzymes: Basic Mechanisms and Health Implications. Physiol. Rev..

[36] Santos D.E., Souza A.D.O., Tibério G.J., Alberici L.C., Hartfelder K. (2020). Differential expression of antioxidant system genes in honey bee (*Apis mellifera* L.) caste development mitigates ROS-mediated oxidative damage in queen larvae. Genet. Mol. Biol..

[37] Raikhel A.S. (1986). Lysosomes in the cessation of vitellogenin secretion by the mosquito fat body; selective degradation of Golgi complexes and secretory granules. Tissue Cell.

[38] Raikhel A.S. (1986). Role of lysosomes in regulating of vitellogenin secretion in the mosquito fat body. J. Insect Physiol..

[39] Dittmer N.T., Raikhel A.S. (1997). Analysis of the mosquito lysosomal aspartic protease gene: An insect housekeeping gene with fat body-enhanced expression. Insect Biochem. Mol. Biol..

[40] Raikhel A.S., Lea A.O. (1983). Previtellogenic development and vitellogenin synthesis in the fat body of a mosquito: An ultrastructural and immunocytochemical study. Tissue Cell.

[41] Nye C.K., Hanson R.W., Kalhan S.C. (2008). Glyceroneogenesis Is the Dominant Pathway for Triglyceride Glycerol Synthesis in Vivo in the Rat. J. Biol. Chem..

[42] Wang T., Geng S.L., Guan Y.M., Xu W.H. (2018). Deacetylation of metabolic enzymes by Sirt2 modulates pyruvate homeostasis to extend insect lifespan. Aging.

[43] He X.J., Barron A.B., Yang L., Chen H., He Y.Z., Zhang L.Z., Huang Q., Wang Z.L., Wu X.B., Yan W.Y. (2022). Extent and complexity of RNA processing in honey bee queen and worker caste development. iScience.

[44] Xu J., Tan A., Palli S.R. (2010). The function of nuclear receptors in regulation of female reproduction and embryogenesis in the red flour beetle, *Tribolium castaneum*. J. Insect Physiol..

[45] Mane-Padros D., Cruz J., Cheng A., Raikhel A.S. (2012). A Critical Role of the Nuclear Receptor HR3 in Regulation of Gonadotrophic Cycles of the Mosquito *Aedes aegypti*. PLoS ONE.

[46] Burmester T., Scheller K. (1999). Ligands and receptors: Common theme in insect storage protein transport. Die Naturwissenschaften.

[47] Braun R.P., Wyatt G.R. (1996). Sequence of the Hexameric Juvenile Hormone-binding Protein from the Hemolymph of *Locusta migratoria*. J. Biol. Chem..

[48] Ismail S.M.U.O., Gillott C. (1995). Identification, characterization, and developmental profile of a high molecular weight, juvenile hormone-binding protein in the hemolymph of the migratory grasshopper, *Melanoplus sanguinipes*. Arch. Insect Biochem. Physiol..

[49] Zhou X., Oi F.M., Scharf M.E. (2006). Social Exploitation of Hexamerin: RNAi Reveals a Major Caste-Regulatory Factor in Termites. Proc. Natl. Acad. Sci. USA.

[50] Martins J.R., Nunes F.M., Cristino A.S., Simoes Z.L., Bitondi M.M. (2010). The four hexamerin genes in the honey bee: Structure, molecular evolution and function deduced from expression patterns in queens, workers and drones. BMC Mol. Biol..

[51] Xiang Y., Liu Z., Huang X. (2010). br regulates the expression of the ecdysone biosynthesis gene npc1. Dev. Biol..

[52] Yamanaka N., Rewitz K.F., O’Connor M.B. (2013). Ecdysone control of developmental transitions: Lessons from *Drosophila* research. Annu. Rev. Entomol..

[53] Moeller M.E., Danielsen E.T., Herder R., O Connor M.B., Rewitz K.F. (2013). Dynamic feedback circuits function as a switch for shaping a maturation-inducing steroid pulse in *Drosophila*. Development.

